# S-Propargyl-Cysteine Attenuates Diabetic Cardiomyopathy in *db/db* Mice Through Activation of Cardiac Insulin Receptor Signaling

**DOI:** 10.3389/fcvm.2021.737191

**Published:** 2021-09-17

**Authors:** Ye Li, Kui-Fang Xie, Ya-Hong Chang, Cheng Wang, Ying Chen, Ming-Jie Wang, Yi-Chun Zhu

**Affiliations:** ^1^Shanghai Key Laboratory of Bioactive Small Molecules and Shanghai Key Laboratory of Clinical Geriatric Medicine, Innovative Research Team of High-Level Local Universities in Shanghai, Department of Physiology and Pathophysiology, School of Basic Medical Sciences, Fudan University, Shanghai, China; ^2^Laboratory Animal Technical Platform, Shanghai Institute of Nutrition and Health, University of Chinese Academy of Sciences, Chinese Academy of Sciences, Shanghai, China

**Keywords:** S-propargyl-cysteine, hydrogen sulfide, diabetic cardiomyopathy, insulin receptor signaling, glucose uptake

## Abstract

**Background:** Endogenous hydrogen sulfide (H_2_S) is emerging as a key signal molecule in the development of diabetic cardiomyopathy. The aim of this study was to explore the effect and underlying mechanism of S-propargyl-cysteine (SPRC), a novel modulator of endogenous H_2_S, on diabetic cardiomyopathy in *db/db* diabetic mice.

**Methods and Results:** Vehicle or SPRC were orally administered to 8-month-old male *db/db* mice and their wild type littermate for 12 weeks. SPRC treatment ameliorated myocardial hypertrophy, fibrosis, and cardiac systolic dysfunction assessed by histopathological examinations and echocardiography. The functional improvement by SPRC was accompanied by a reduction in myocardial lipid accumulation and ameliorated plasma lipid profiles. SPRC treatment improved glucose tolerance in *db/db* mice, with fasting blood glucose and peripheral insulin resistance remaining unchanged. Furthermore, insulin receptor signaling involving the phosphorylation of protein kinase B (Akt/PKB) and glycogen synthase kinase 3β (GSK3β) were elevated and activated by SPRC treatment. Primary neonatal mice cardiomyocytes were cultured to explore the mechanisms of SPRC on diabetic cardiomyopathy *in vitro*. Consistent with the results *in vivo*, SPRC not only up-regulated insulin receptor signaling pathway in cardiomyocytes in dose-dependent manner in the basal state, but also relieved the suppression of insulin receptor signaling induced by high concentrations of glucose and insulin. Furthermore, SPRC also enhanced the expression of glucose transporter 4 (GLUT4) and ^3^H glucose uptake in cardiomyocytes.

**Conclusions:** In this study, we found a novel beneficial effect of SPRC on diabetic cardiomyopathy, which was associated with activation of insulin receptor signaling. SPRC may be a promising medication for diabetic cardiomyopathy in type 2 diabetes mellitus patients.

## Introduction

Among adults in China, the number of diabetic patients have explosively increased. The estimated overall prevalence of diabetes was 10.9%, and that for prediabetes was 35.7% (1). Cardiovascular disease, is a leading cause of mortality in the development of diabetic complications (2). After adjusting for other risk factors including age, hypertension, obesity, dyslipidemia, the incidence of heart failure increases 2.4–5 fold in diabetic patients than non-diabetic patients (3). Diabetic cardiomyopathy (DCM) is defined as structural and functional abnormalities in the myocardium of diabetic patients independent of underlying coronary artery disease and hypertension (4). At present, treatment strategy for DCM mainly rely on conventional therapies that focus on optimizing glycemic control (5). However, meta-analysis of large clinical trials revealed that strict glycemic control had no impact on the incidence of heart failure in diabetic patients (6, 7). Therefore, it is particularly important to explore the pathogenesis mechanism and novel therapeutic drugs of DCM.

Hydrogen sulfide (H_2_S) has been considered toxic and odorous for a long time. However, it has been recognized as the third gasotransmitter following nitric oxide and carbon monoxide to play an important role in many physiological and pathological processes since the 1990s. Accumulating studies have shown that H_2_S is involved in improving DCM by multiple mechanisms (8), such as supplementation of exogenous H_2_S reduced endoplasmic reticulum pressure in cardiomyocytes (9, 10) and inhibited myocardial oxidative Stress, inflammation, and apoptosis (11). Endogenous H_2_S production by cystathionine-γ-lyase (CTH, also named CSE, the main producing enzyme of endogenous H_2_S in cardiovascular system) is inhibited in myocardium of DCM rats (12). Consistently, H_2_S levels in serum of DCM patients were significant decreased and the supplement of H_2_S could rescue the cardiomyopathy dysfunction induced by hyperglycemia (10, 13). However, H_2_S cannot be used for clinical therapy because of its instability and gaseous characteristics. S-Propargyl-Cysteine (SPRC, also named as ZYZ-802), a novel water-soluble modulator of endogenous H_2_S, promotes the activity of CSE and then increases H_2_S levels in plasma or tissue (14–16). In this study, we evaluated the effect of SPRC on DCM in *db/db* mice and further explored the underlying mechanism, both *in vivo and in vitro*.

## Materials and Methods

### Chemicals

S-Propargyl-Cysteine (SPRC, purity > 99%) was synthesized as described previously (17) and provided by Professor Zhu Yi-Zhun (State Key Laboratory of Quality Research in Chinese Medicine and School of Pharmacy, Macau University of Science and Technology, Macau, China). Chemical formula of SPRC is shown in Supplementary Figure 1A.

### Animals and Treatments

All experimental procedures were performed in accordance with Guide for the Care and Use of Laboratory Animals of the National Institutes of Health (NIH) of the United States and approved by the Ethics Committee of Experimental Research, Fudan University Shanghai Medical College. Male C57BLKS/J *db/db* mice and their wild type littermates (7-week-old) were purchased from Nanjing Biomedical Research Institute of Nanjing University and were housed in a climate-controlled environment (22 ± 2°C, 45–75% relative humidity) with a 12 h light-dark cycle and unrestricted access to food and water. After acclimatization for 1 weeks, wild-type (WT) mice were used as a normal control group and *db/db* mice were randomly divided into four different groups (*n* = 15–16 per group): diabetic model group, low-dose SPRC treatment group (20 mg/kg per day), medium-dose SPRC treatment group (40 mg/kg per day), and high-dose SPRC treatment group (80 mg/kg per day). Both WT control group and diabetic model group were orally administered an equal volume of vehicle (ddH_2_O). After 12 weeks of administration, mice were anesthetized by intraperitoneal injection of 1% sodium pentobarbital and blood samples were collected. Heart weights (HW) and tibia lengths (TL) were measured and heart tissue specimens were obtained.

### Transthoracic Echocardiography

At the first and last week of SPRC treatment, the hair was removed from the chest of mice using depilatory cream. The mice were then anesthetized with 1.5% isoflurane and placed in a supine position on the test bench, with ultrasound gel applied onto the chest. Mouse two-dimensional echocardiography was performed using a Vevo3100 ultrasound device (VisualSonics Inc., Canada), as previously described (18). B-mode, M-mode, and Power Doppler Mode ultrasound images of the left ventricle were recorded. All measurements were averaged for five consecutive cardiac cycles.

### Fasting Blood Glucose, Body Weight, and Fasting Plasma Insulin Levels

The FBG levels and BW of mice were monitored every 2 weeks after food was removed for 12 h. Glucose measurements were performed with venous blood collected from mice tails by glucose monitors (ONETOUCH, Johnson and Johnson, USA). After food was removed overnight for 16 h, FINS were detected by enzyme-linked immunosorbent assay (ELISA) kit (Mercodia, Sweden). The formula of homeostasis model assessment for insulin resistance index (HOMA-IR) is [(HOMA-IR) = (FBG × FINS)/22.5] (19).

### IPGTTs and IPITTs

Intraperitoneal glucose tolerance test (IPGTT) and intraperitoneal insulin tolerance test (IPITT) were performed in the morning on nonfasted mice that had their food removed 1 h prior to intraperitoneal injection (IPGTT 1 g glucose per kg body weight and IPITT 1unit recombinant human insulin (Humulin 70/30, Lilly, USA) per kg body weight), Blood glucose levels were measured before the injection (time 0) and 15, 30, 60, and 120 min after the injection. Areas under the curve (AUC) were determined using the trapezoidal rule.

### Biochemical Analyses

The total cholesterol (TC), triglycerides (TG), high-density lipoprotein cholesterol (HDL-C), low-density lipoprotein cholesterol (LDL-C), non-esterified fatty acid (NEFA) in plasma were determined using commercial kits (Nanjing Jiancheng, China) according to the manufacturer's instructions. Briefly, full-wavelength microplate reader (Infinite® 200 PRO, TECAN, Switzerland) was used to detect the absorbance of plasma samples at 510 nm for TC and TG or at 546 nm for HDL-C, LDL-C and NEFA.

### Histological Analysis

Heart specimens were fixed in 4% paraformaldehyde solution. After being embedded in paraffin, the specimens were cut into 5-μm-thick sections and stained with wheat germ agglutinin (WGA) and Masson's trichrome. The other heart tissue specimens were frozen and stained with Oil Red O lipid stain. The size of myocardial cells, fiber area fraction, and myocardial lipid content were determined using Image J software (Bethesda, MA, USA).

### Transmission Electron Microscopy

To observe myocardial ultrastructure, heart tissues were cut into 1 mm transverse sections and immersed in 2% glutaraldehyde overnight. The sections were then immersed in 1% osmium tetroxide for 2 h, dehydrated in graded ethanol, and embedded in epoxy resin. Ultrathin sections (60–70 nm) were obtained, stained with uranyl acetate and lead citrate, and examined using a Tecnai G20 Twin transmission electron microscope (FEI, USA).

### Primary Culture of Neonatal Mice Cardiomyocytes

Primary cultures of cardiomyocytes were prepared from neonatal mice hearts. In brief, hearts were excised from 1- to 2-day-old mice pups. Ventricles were minced by small scissors and digested using 1 mg/ml collagenase type II (Worthington, USA). The digested solution was collected. The process was repeated 3–4 times until no chunks of tissue were visible. The final pooled solution was centrifuged and the cell pellet re-suspended in Dulbecco's Modified Eagle Medium (DMEM, Hyclone, USA), high Glucose containing 10% fetal bovine serum (FBS, Gibco, USA). The cells were pre-plated for 1 h to allow the attachment and removal of fibroblasts. The unattached cardiomyocytes remaining in suspension were then collected and plated in DMEM, high Glucose containing 10% FBS. After 48 h Cardiomyocytes cultures were used for subsequent experiments.

### Cell Viability

Cell viability was determined by cell counting kit-8 assay according to manufacturer's instructions (DOJINDO, Japan). Cardiomyocytes were cultured in a 96-well-culture plate and treated with different concentrations of SPRC (0–1,000 μM) for 24 h. Cells were subsequently incubated with 10 ml CCK-8 solution at 37°C for 4 h. The absorbance at 450 nm was measured.

### Real-Time PCR

Total RNA was extracted by Trizol reagent from heart tissue or cardiomyocytes. RNA was reverse-transcribed using a cDNA synthesis kit (Toyobo Life Science, Japan). Real-Time PCR was performed using a StepOnePlus Real-Time PCR Detection System (Applied Biosystems Inc., CA, USA). A total volume of 20 μL reaction mix containing 2 μL cDNA, 10 μL SYBR Green PCR Master Mix (Toyobo, Japan) and 1 μL each primer (10 μM). G*apdh* was used for normalization and the relative expression of mRNA was calculated according to the ^ΔΔ^Ct method. The specific primers were as follows: *Myh7*: 5′-ATCAATGCAACCCTGGAGAC-3′, 5′-CGAACATGTGGTGGTTGAAG-3′; *InsR*: 5′-GCTTCTGCCAAGACCTTCAC-3′, 5′-CACTCGGGGATGCACTTATT-3′; *Glut4*: 5′-ACCCTGGGCTCTGTATCCC-3′, 5′-CCCTGACCACTGAGTGCAAA-3′; *Gapdh:* 5′-TTCACCACCATGGAGAAGGC-3′, 5′-GGCATGGACTGTGGTCATGA-3′; β*-actin:* 5′- GACAGGATGCAGAAGGAGATTACT-3′, TGATCCACATCTGCTGGAAGGT-3′.

### Western Blotting

Left ventricular tissue and cell samples were lysed with Cell Lysis Buffer for Western (P0031; Beyotime Biotechnology, China) containing a protease inhibitor cocktail (049693132001; Roche, Basel, Switzerland). The crude cell lysate was centrifuged and the supernatant was harvested. The concentration of protein in the supernatant was quantified by standard bicinchoninic acid assay (BCA, P0012, Beyotime Biotechnology, China). For western blotting, equal amounts of proteins were resolved by sodium dodecyl sulfate polyacrylamide gel electrophoresis and transferred to polyvinylidene difluoride membranes. Membranes were then incubated with horseradish peroxidase-conjugated secondary antibodies (1:10,000; A0208; Beyotime Biotechnology, China) for 2 h, and immunoreactive bands were visualized by chemiluminescence. And the gray value of the captured bands was determined by ImageJ software. The primary antibodies used included: anti-Insulin Receptor β (#3025), anti-Phospho-Insulin Receptor β Tyr1150/1151(#3024), anti-Akt (#9272), anti-phospho-Akt Ser473 (#4060), anti-GSK-3β (#9315), anti-Phospho-GSK-3β Ser9 (#5558) were purchased from Cell Signaling Technology (Beverly, MA, USA); anti-GAPDH (60004-1), anti-β-Actin (66009-1), and anti-α-Tubulin (11224-1) purchased from Proteintech Group (Chicago, USA), and anti-CSE(sc-374249) were purchased from Santa Cruz Biotechnology (Texas, USA).

### 2-Deoxyglucose Uptake

Primary mice cardiomyocytes were incubated with 33.3 mM glucose (HG), 100 nM insulin (HI), or 33.3 mM glucose and 100 nM insulin (HG++HI) for 48 h. Among them, the HG+HI group treated with vehicle or different doses of SPRC (25–100 μM) for 24 h. then the uptake of 2-deoxyglucose by cardiomyocytes was measured as previously described (20). The cardiomyocytes were rinsed with pre-warmed KRP buffer (128 mM NaCl, 4.7 mM KCl, 1.25 mM CaCl_2_, 1.25 mM MgSO_4_, 5 mM NaH_2_PO_4_, 5 mM Na_2_HPO_4_, and 10 mM HEPES, pH 7.4) for three times and then treated with 100 nM insulin in KRP buffer without glucose for 30 min in 37°C. Subsequently, the cardiomyocytes were incubated with 2-deoxy-D[^3^H]-glucose (1 μCi/ml) dissolved in KRP buffer with 100 nM insulin for 10 min in 37°C. After washing with pre-cold phosphate-buffered saline (PBS) containing 10 mM glucose for 3 times, the cells were lysed with 0.4 mM NaOH for 2 h. The radioactivity of ^3^H was measured in a liquid scintillation counter (Beckman LS6500) and the concentration of protein were detected by BCA method for standardization.

### Measurement of H_2_S Levels

H_2_S levels in heart tissues and plasma were measured as previously described (21). Briefly, heart tissues were homogenized in Tris-HCl (100 mmol/L, pH 8.5), then centrifuged at 12,000 g for 20 min at 4°C. The plasma sample were directly centrifuged at 3,500 rpm for 15 min at 4°C. And 30 μl of the supernatant from heart tissues and plasma were derivatized by MBB and detected by HPLC-MS. The concentration of protein from heart tissues were detected by BCA method for standardization.

### Statistical Analysis

All data were expressed as the means ± SEM from at least three independent experiments. SPSS 13.0 software (SPSS, Inc., Chicago, USA) was used for statistical analysis. Student's unpaired *t*-test was performed to statistically analyze individual group data. Multiple-group comparisons were evaluated by one-way analysis of variance (ANOVA) followed by Fisher's Least Significant Difference (LSD) and the non-parametric test was used when the variance was not equal. Values of *P* < 0.05 were considered to be statistically significant.

## Results

### SPRC Treatment Improves Cardiac Function, and Alleviates Myocardial Hypertrophy and Fibrosis on Diabetic Cardiomyopathy

**Figure 1:**
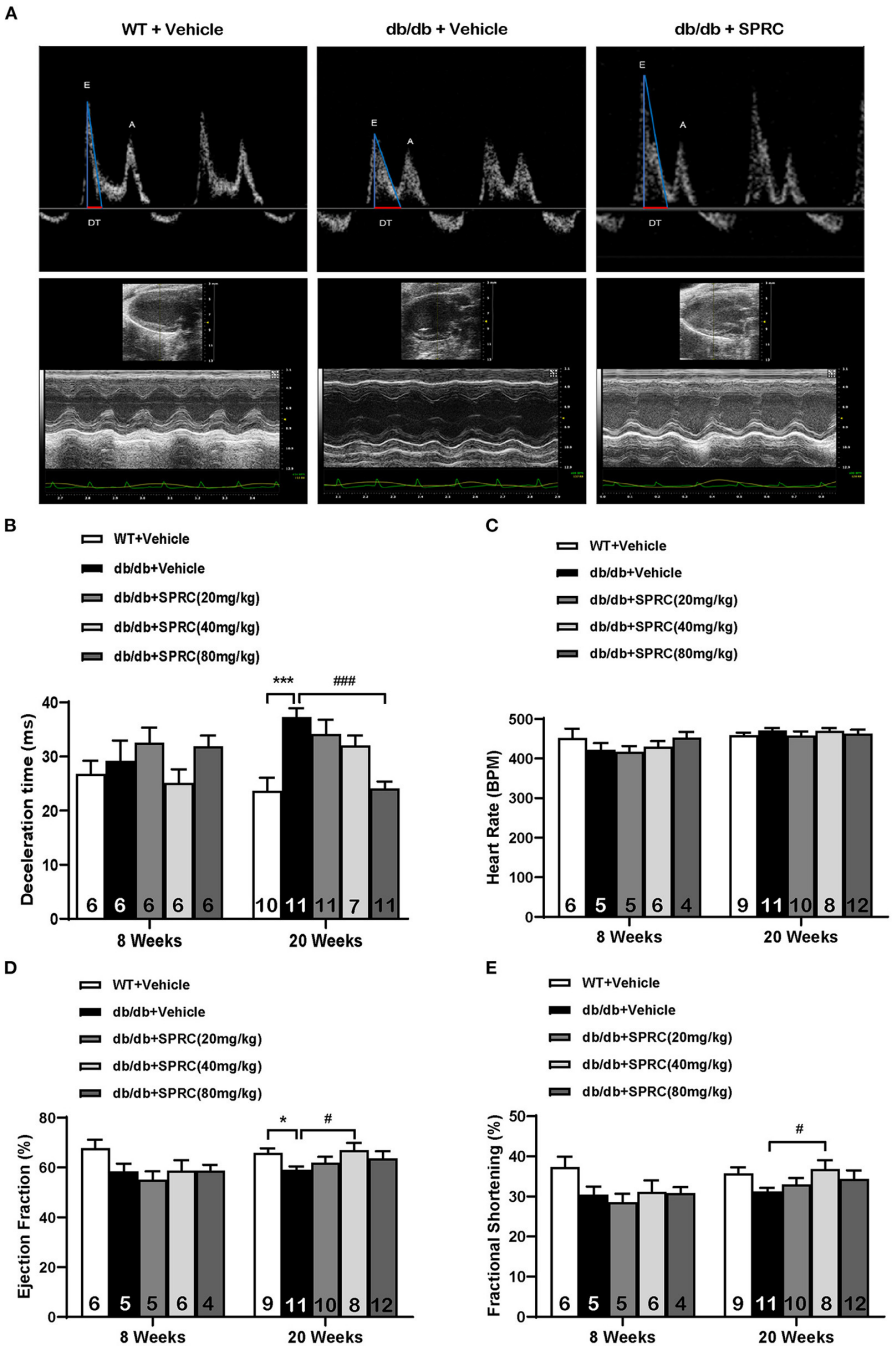
SPRC treatment improves cardiac function in *db/db* mice. Vehicle or SPRC (20, 40, or 80 mg/kg/day) was orally administered to WT mice or *db/db* mice for 12 weeks. Echocardiograph images were recorded before and after 12 weeks of SPRC treatment. **(A)** Representative Power Doppler Mode (upper) and M-Mode (lower) echocardiograph images. **(B–E)** Echocardiographic assessment of deceleration time (DT), heart rate (HR), left ventricular ejection fraction (EF), and fractional shortening (FS). Values are presented as means ± SEM (*n* = 4–12). *P < 0.05, ***P < 0.001 vs. WT + Vehicle group, ^#^*P* < 0.05, ^*###*^*P* < 0.001 vs. *db/db* + Vehicle mice.

**Figure 2:**
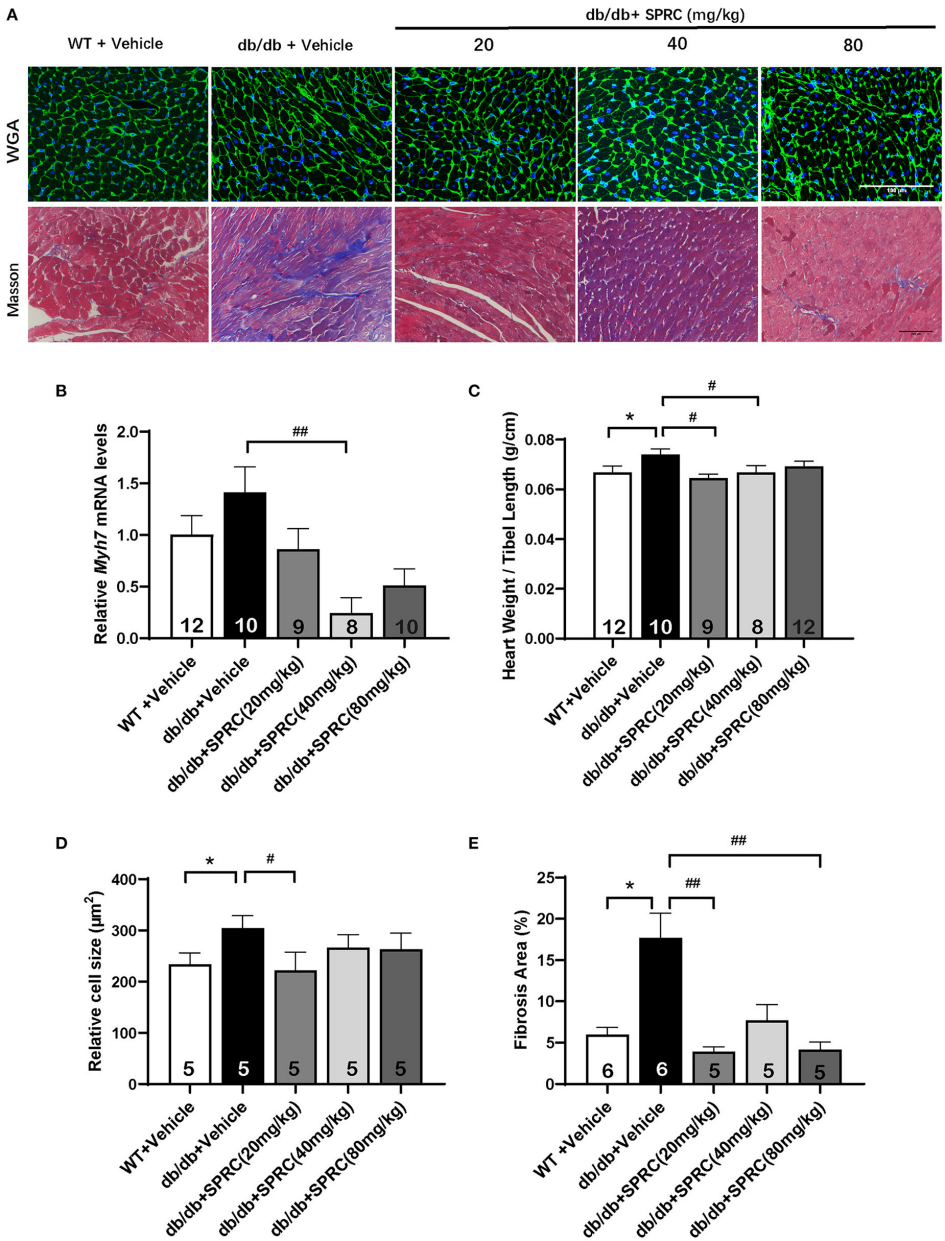
SPRC treatment improves myocardial hypertrophy and fibrosis in *db/db* mice. Vehicle or SPRC (20, 40, or 80 mg/kg/day) was orally administered to WT mice or *db/db* mice for 12 weeks. Heart tissue specimens were collected. **(A)** Wheat germ agglutinin (WGA) and masson staining of myocardium were performed. **(B)** mRNA levels of *Myh7* in heart tissue were determined by real-time qPCR. mRNA levels of *Gapdh* was used as reference for normalization (*n* = 8–12). **(C)** The ratio of heart weights (HW) and tibia lengths (TL) was calculated (*n* = 8–12). Average cell area (*n* = 5) **(D)** and fibrosis area (*n* = 5–6) **(E)** were shown. Values are presented as means ± SEM. *P < 0.05 vs. WT + Vehicle group, ^#^*P* < 0.05, ^*##*^*P* < 0.01 vs. *db/db* + Vehicle mice.

To explore the effect of SPRC on cardiac function of *db/db* diabetic mice, echocardiograph images were recorded before and after 12 weeks of SPRC treatment (Figure 1A). Compared with WT littermates, *db/db* mice preserved cardiac function at 8-week-old, but developed diastolic and systolic dysfunction at 20-week-old characterized by elevated deceleration time (DT, Figure 1B), reduced left ventricular ejection fraction (EF, Figure 1D) and decreased fractional shortening (FS, Figure 1E, *P* = 0.054) with a consistent heart rate (HR, Figure 1C). All of three parameters examined were improved by SPRC treatment (Supplementary Tables 1, 2). The beneficial effect of SPRC treatment on cardiac hypertrophy was confirmed by histopathological examinations and RT-PCR (Figure 2). Heart weight to tibia length (HW/TL) ratio was significantly increased in *db/db* mice compared with WT mice (Figure 2C). Consistently, cardiomyocyte cross-sectional area examined in *db/db* mice by wheat germ agglutinin (WGA) was markedly enlarged (Figure 2D). Administration of SPRC to *db/db* mice decreased HW/TL ratios and cardiomyocyte cross-sectional area, reduced mRNA level of beta-myosin heavy chain (Myh7) gene, marker for cardiac hypertrophy (Figure 2B). Masson staining revealed that obvious fibrosis in the interstitial of myocardium was observed in *db/db* mice, but significantly alleviated by SPRC treatment (Figure 2E). These data suggest that SPRC treatment improves cardiac function, and alleviates cardiac hypertrophy and fibrosis in the *db/db* diabetic model.

### SPRC Treatment Alleviated Abnormal Myocardial Ultrastructure on Diabetic Cardiomyopathy

**Figure 3:**
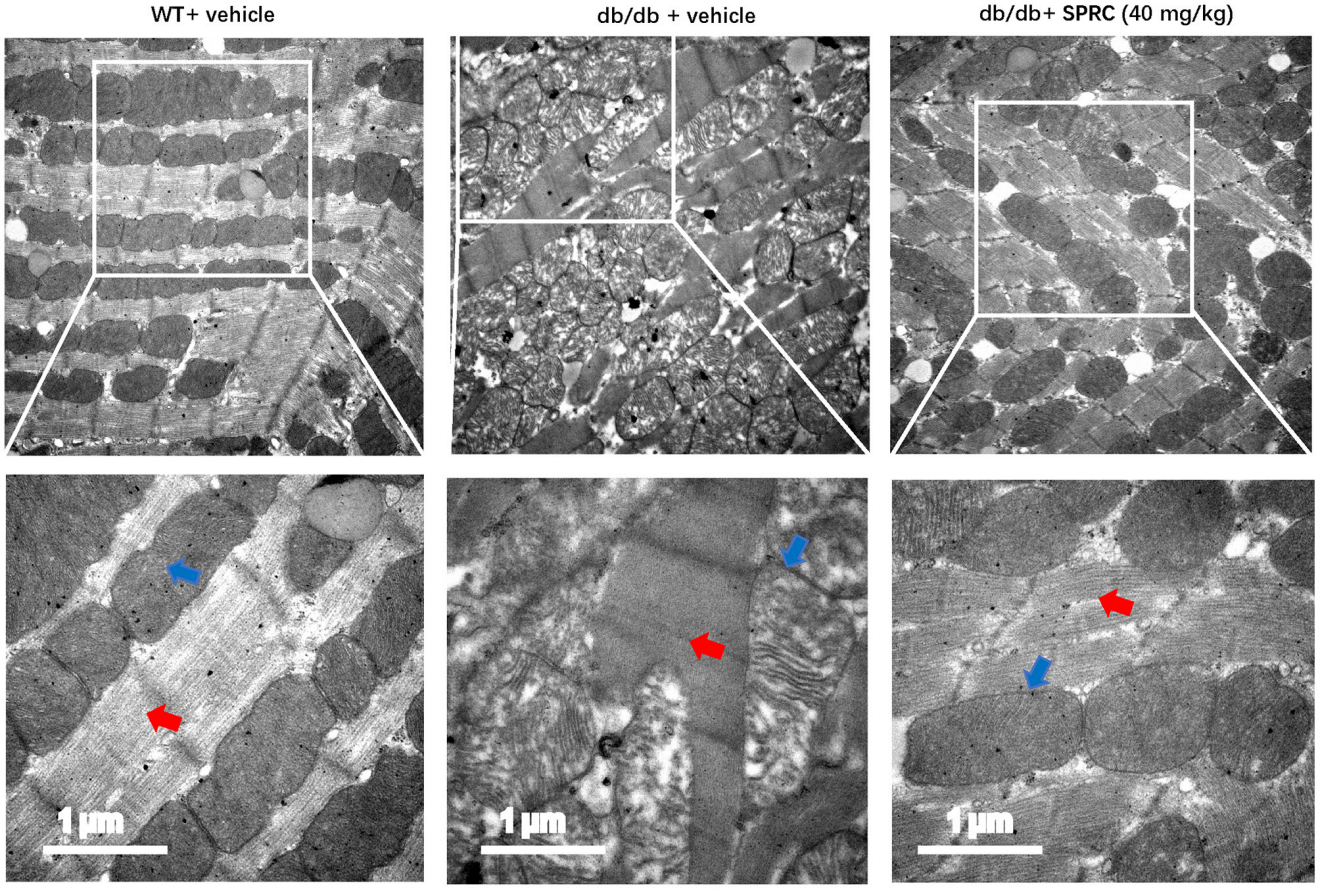
SPRC treatment improves abnormal ultrastructure of cardiomyocytes in *db/db* mice. Vehicle or SPRC (20,40, or 80 mg/kg/day) was orally administered to WT mice or *db/db* mice for 12 weeks. Heart tissue specimens were collected and representative images of the ultrastructural morphology of cardiomyocytes were shown. The red arrow indicates myofilament and the blue arrow indicates mitochondria (*n* = 2).

SPRC treatment protected *db/db* mice from abnormalities in the ultrastructure of cardiomyocytes. The mitochondria in the cardiomyocytes of WT mice were regularly arranged and exerted integral cristae, and the myofilaments were arranged tightly, while a portion of mitochondria in the myocardium of *db/db* mice were swollen, disorderly arranged with broken cristae, and the myofilaments were loose. After SPRC treatment, fewer abnormal ultrastructure of mitochondria and myofilaments were observed in diabetic mice (Figure 3).

### SPRC Treatment Reduces Myocardial Lipid Accumulation and Dyslipidemia on Diabetic Cardiomyopathy

**Figure 4:**
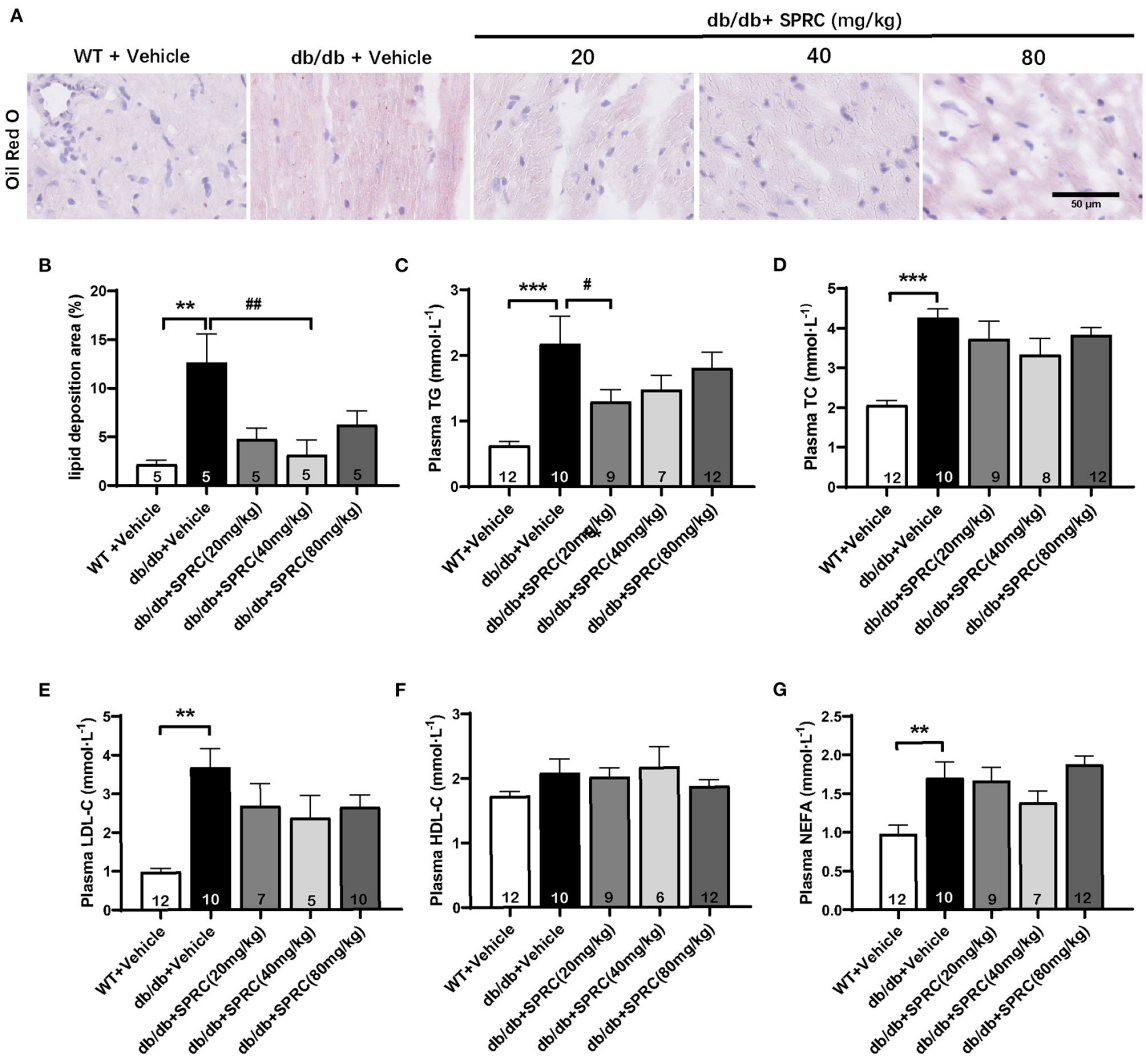
SPRC treatment reduces myocardial lipid accumulation and dyslipidemia in *db/db* mice. Vehicle or SPRC (20, 40, or 80 mg/kg/day) was orally administered to WT mice or *db/db* mice for 12 weeks. Heart tissue specimens and plasma were collected. **(A)** Oil Red O staining of myocardium were performed (*n* = 5) lipid deposition area **(B)** was shown. The levels of TC **(C)**, TG **(D)**, LDL-C **(E)**, HDL-C **(F)**, NEFA **(G)** were measured (*n* = 5–12). Values are presented as means ± SEM. **P < 0.01, and ***P < 0.001 vs. WT + Vehicle group, ^#^*P* < 0.05, ^*##*^*P* < 0.01 vs. *db/db* + Vehicle mice.

Oil red O staining showed a predictable and significantly increase in lipid droplets of *db/db* hearts, which was markedly decreased in response to SPRC (Figures 4A,B). Meanwhile, systemic dyslipidemia was observed in *db/db* mice characterized by markedly increased TC, TG, LDL-C, and NEFA. SPRC treatment inhibited the increased plasma TC levels, indicating that SPRC has a certain lipid-lowering effect (Figures 4C–G).

### SPRC Treatment Improves Glucose Tolerance in *db/db* Mice, With Fasting Blood Glucose and Systemic Insulin Resistance Remaining Unchanged

**Figure 5:**
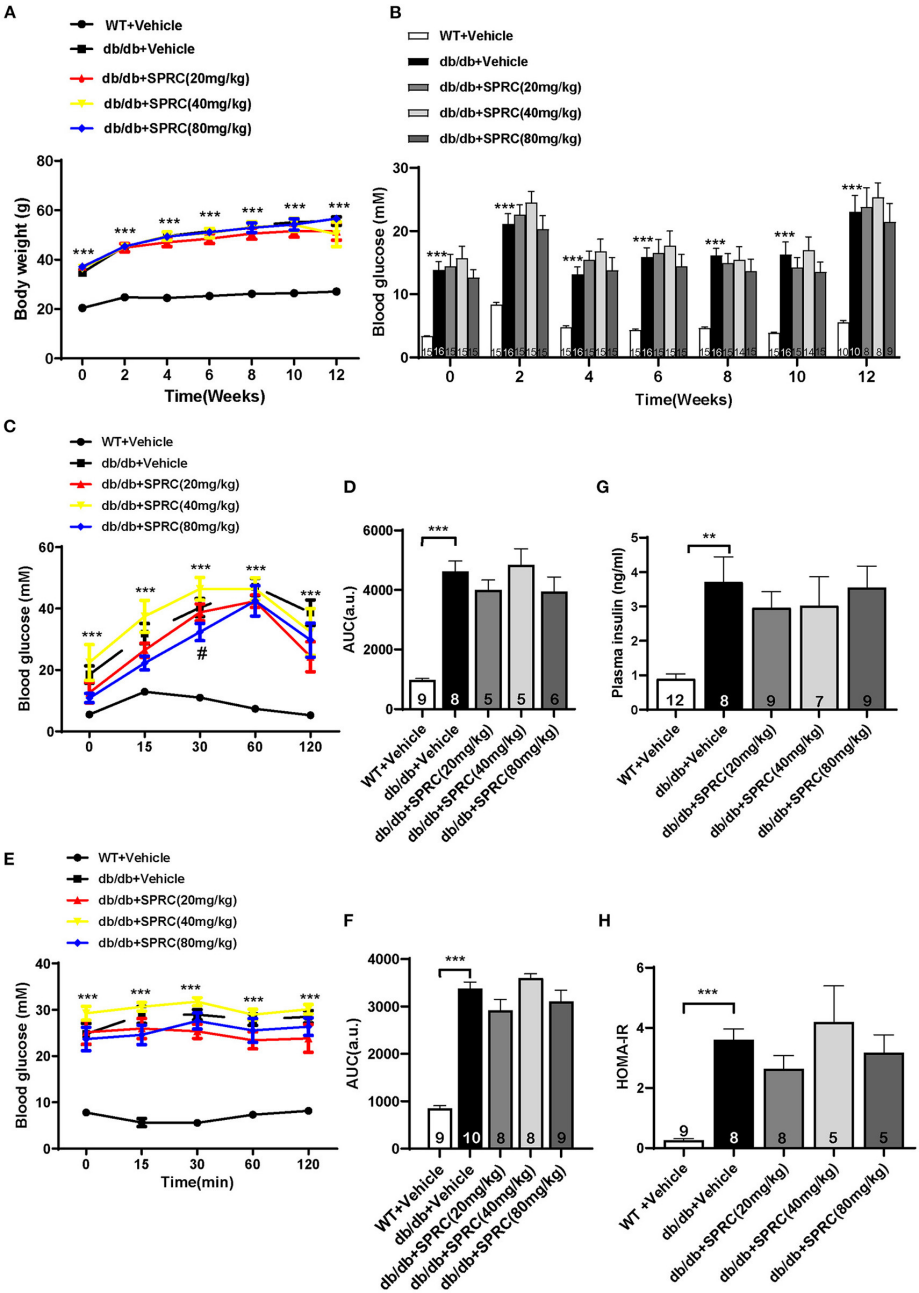
SPRC treatment improves glucose tolerance in *db/db* mice, with fasting blood glucose and systemic insulin resistance remaining unchanged. Vehicle or SPRC (20, 40, or 80 mg/kg/day) was orally administered to WT mice or db/db mice for 12 weeks. **(A)** Body weight (*n* = 8–16), **(B)** fasting blood glucose (*n* = 8–16), **(C)** intraperitoneal glucose tolerance test (IPGTT) and **(D)** area under curve (AUC) of IPGTT (*n* = 5–9), **(E)** intraperitoneal insulin tolerance test (IPITT) and **(F)** area under curve (AUC) of IPITT (*n* = 8–10), **(G)** fasting plasma insulin (*n* = 7–12), and **(H)** HOMA-IR index were determined (*n* = 5–9). Values are presented as means ± SEM. **P < 0.01, and ***P < 0.001 vs. WT + Vehicle group, ^#^*P* < 0.05 vs. *db/db* + Vehicle mice.

Throughout the experiment, the body weight and fasting blood glucose of *db/db* mice were significantly increased compared to their WT littermates. However, SPRC treatment showed no significant impact on body weight and blood glucose (Figures 5A,B). To investigate the effect of SPRC on systemic insulin resistance, IPGTT and IPITT were preformed and the results demonstrated SPRC treatment at a dosing of 80 mg·kg^−1^·day^−1^ improved glucose tolerance to a certain extent, but has no significant impact on IPITT and its area under curve (Figures 5C–F). Furthermore, fasting plasma insulin levels and HOMA-IR index were remarkably elevated, with SPRC treatment failed to alleviate (Figures 5G,H).

### SPRC Treatment Activated Insulin Receptor Signaling in Both Primary Mice Cardiomyocytes and Myocardium of *db/db* Mice

**Figure 6:**
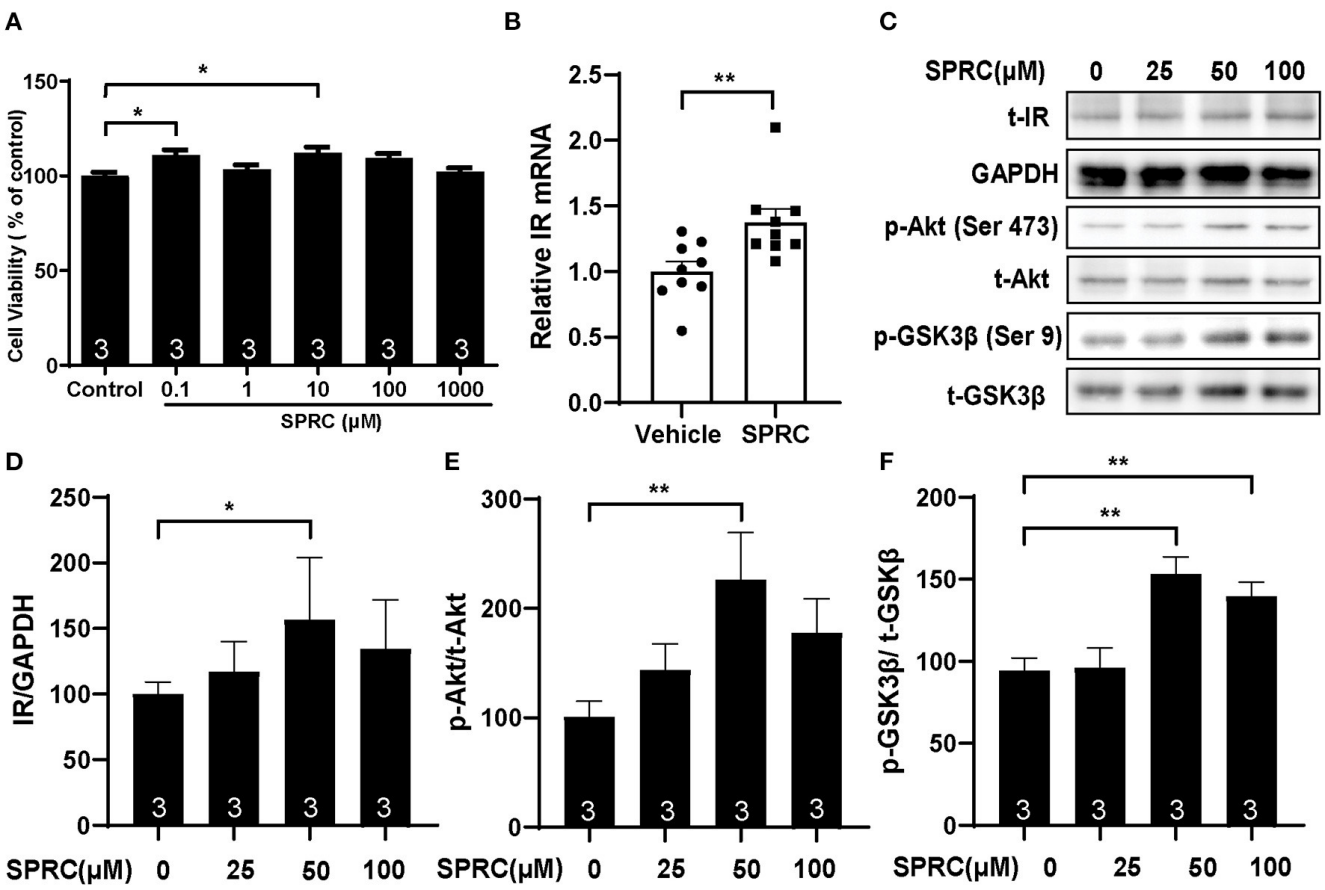
SPRC treatment activated cardiac insulin receptor signaling in primary cardiomyocytes. **(A)** Primary mice cardiomyocytes were exposed to various concentrations of SPRC (0.1–1,000 μM) for 24 h. Cell viability was determined (*n* = 3). *P < 0.05 vs. control group. **(B)** Primary mice cardiomyocytes were treated with 50 μM SPRC for 24 h. mRNA levels of insulin receptor (*Insr*) were determined by real-time qPCR. mRNA levels of *Gapdh* was used as reference for normalization (*n* = 3). **P < 0.01 vs. Vehicle group. **(C–F)** Primary mice cardiomyocytes were treated with vehicle or SPRC (25, 50, and 100 μM) for 24 h. The expression level of protein in insulin receptor signaling were determined by western blotting (*n* = 3). *P < 0.05, **P < 0.01 vs. Vehicle. Values are presented as means ± SEM.

Primary neonatal mice cardiomyocytes were cultured and identified to explore the mechanisms of SPRC on diabetic cardiomyopathy *in vitro* (Supplementary Figure 1B). Incubation of cardiomyocytes with SPRC (0.1–1,000 μM) for 24 h had no detectable cytotoxicity. Moreover, the cell viability was significantly increased at doses of 0.1 and 10 μM of SPRC treatment (Figure 6A). SPRC improved both mRNA and protein expression level of IR in cardiomyocytes, with the classical downstream pathway protein kinase B (Akt/PKB) and glycogen synthase kinase 3β (GSK3β) activated in the dose-dependently manner (Figures 6B–F).

**Figure 7:**
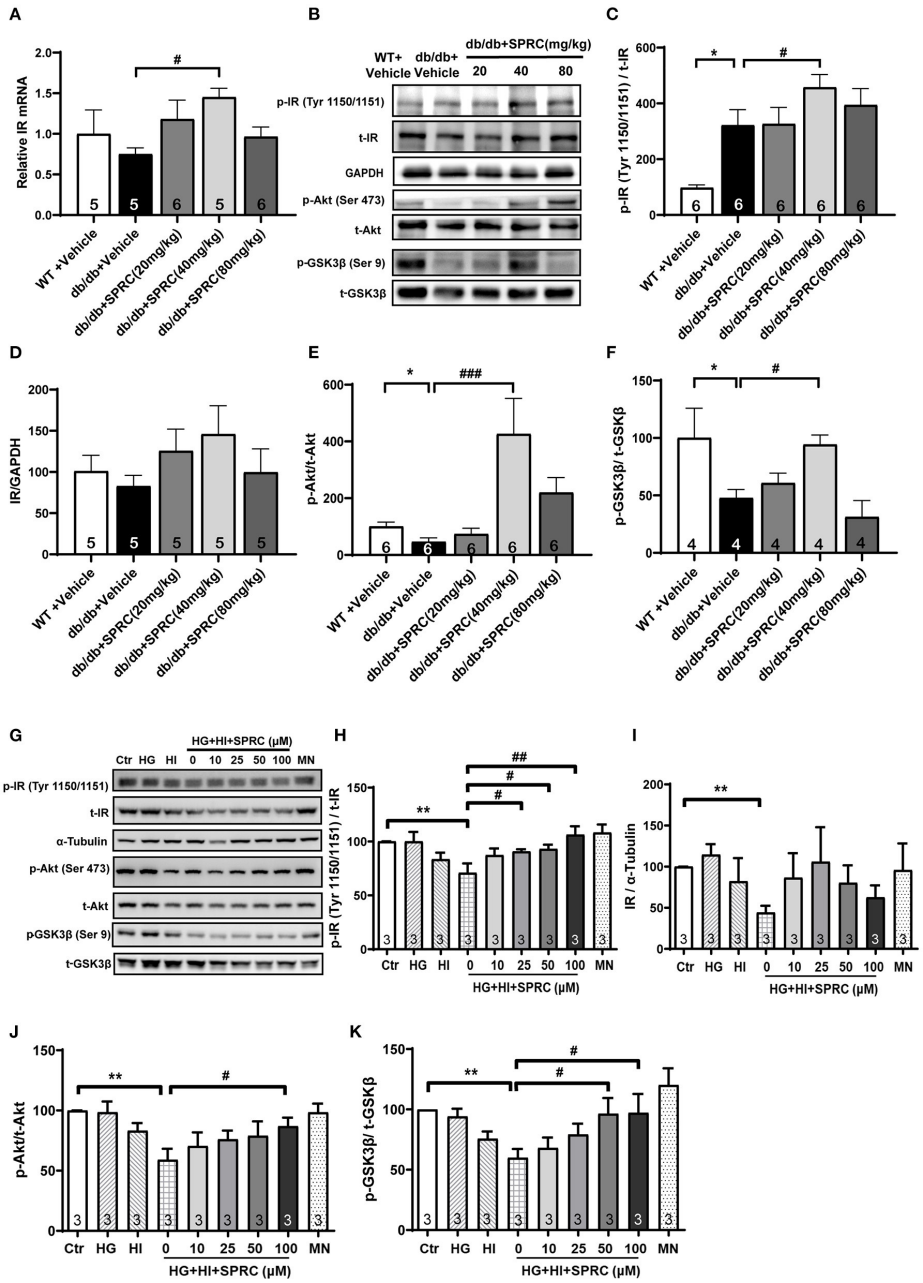
SPRC treatment activated insulin receptor signaling in *db/db* mice. Vehicle or SPRC (20, 40, or 80 mg/kg/day) was orally administered to WT mice or *db/db* mice for 12 weeks. **(A)** The mRNA levels of insulin receptor (*Insr*) were determined by real-time qPCR. mRNA levels of *Gapdh* was used as reference for normalization (*n* = 5–6). ^#^*P* < 0.05 vs. *db/db* + Vehicle mice. **(B–F)** the expression level of protein in insulin receptor signaling were determined by western blotting. *P < 0.05, **P < 0.01, and ***P < 0.001 vs. WT + Vehicle group, ^#^*P* < 0.05, ^*##*^*P* < 0.01, and ^*###*^*P* < 0.001 vs. *db/db* + Vehicle mice. **(G–K)** Primary mice cardiomyocytes were incubated with 33.3 mM glucose (HG), 100 nM insulin (HI), or 33.3 mM glucose + 100 nM insulin (HG + HI) for 48 h. The cardiomyocytes were then incubation with SPRC (50 μM) for 24 h. Expression of insulin receptor signaling were determined (*n* = 3). *P < 0.05, **P < 0.01 < 0.001 vs. control group, ^#^*P* < 0.05, ^*##*^*P* < 0.01, and ^*###*^*P* < 0.001 vs. HG + HI group. Values are presented as means ± SEM.

The cardiac inulin receptor signaling *in vivo* were also determined. As shown in Figures 7A–D, SPRC treatment increased the mRNA of IR in myocardium, and it also improved protein expression of IR to a certain extent (*P* = 0.095). It is noteworthy that IR in the myocardium of diabetic mice was significantly phosphorylated, which is exacerbated by SPRC treatment. The phosphorylation levels of Akt and GSK3β in myocardium of *db/db* mice were significantly decreased, SPRC (80 mg/kg) significantly increased the phosphorylation levels of Akt and GSK3β in myocardium of DCM mice (Figures 7E,F).

We then exposed neonatal mice cardiomyocytes to 33.3 mM glucose and 100 nM insulin to mimic hyperglycemia and hyperinsulinemia in cardiomyocyte of diabetic heart. High concentrations of glucose and insulin stimulation significantly reduced the phosphorylation levels of Akt and GSK3β and the expression level of IR. However, after incubation with 50 μM SPRC for 24 h, the phosphorylation level of IR was preserved, and the expression level of IR was partly increased without statistical significance (*P* = 0.077). Consistently, the downstream of IR signaling including the phosphorylation levels of Akt and GSK3β were preserved (Figures 7G–K).

### SPRC Treatment Increased GLUT4 Expression in Myocardium and Enhanced ^3^H Glucose Uptake in Cultured Primary Cardiomyocytes

**Figure 8:**
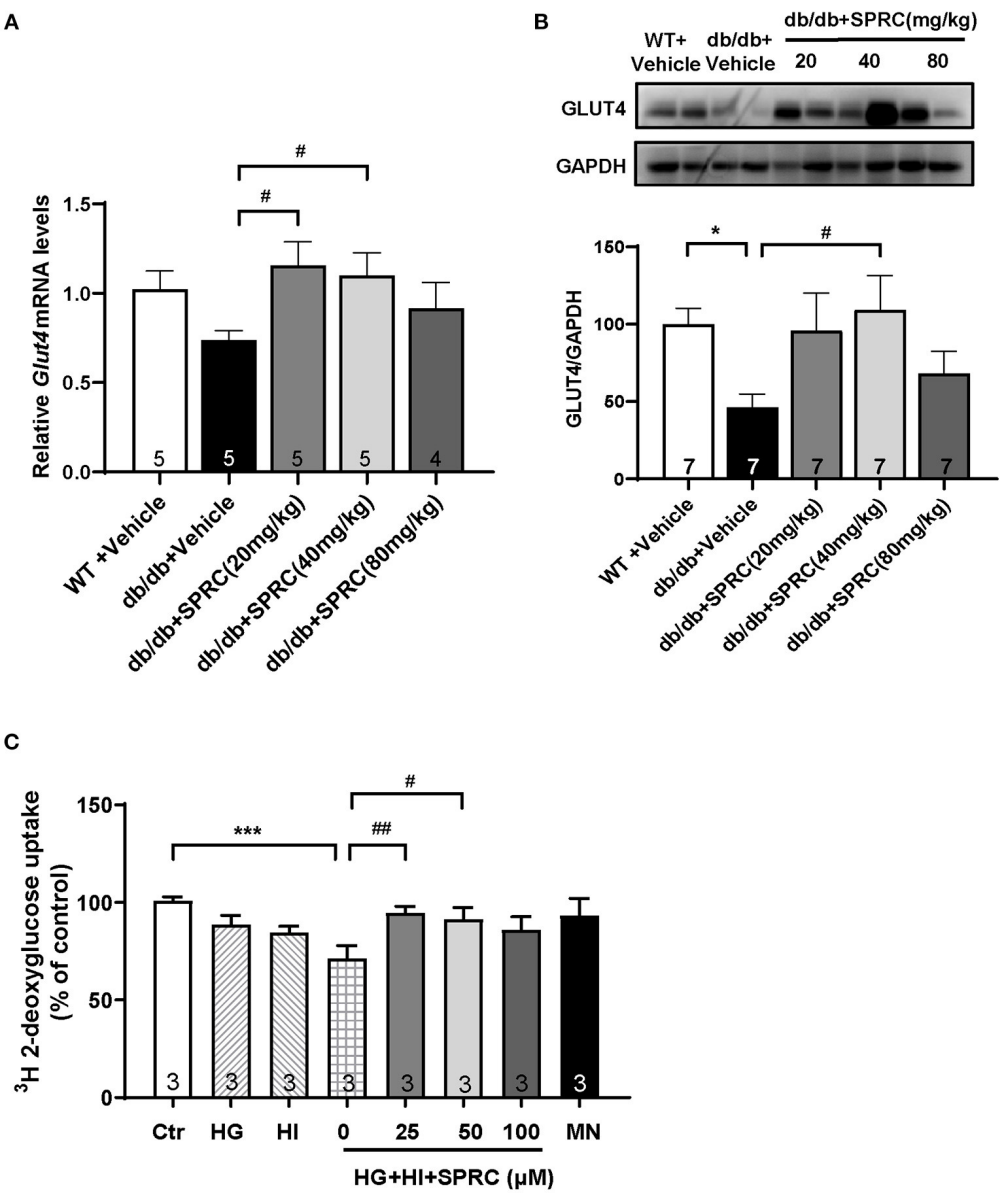
SPRC treatment increased GLUT4 expression and enhanced ^3^H glucose uptake. Vehicle or SPRC (20, 40, or 80 mg/kg/day) was orally administered to WT mice or *db/db* mice for 12 weeks. **(A)** The mRNA levels of *Glut4* were determined by real-time qPCR, and the mRNA levels of *Gapdh* was used as reference for normalization (*n* = 4–5). ^#^*P* < 0.05 vs. *db/db* + Vehicle mice. **(B)** The expression level of *Glut4* in the myocardium were determined by western blotting (*n* = 7). *P < 0.05, **P < 0.01, and ***P < 0.001 vs. WT + Vehicle group, ^#^*P* < 0.05, ^*##*^*P* < 0.01, and ^*###*^*P* < 0.001 vs. *db/db* + Vehicle mice. **(C)** Primary mice cardiomyocytes were incubated with 33.3 mM glucose (HG), 100 nM insulin (HI), or 33.3 mM glucose + 100 nM insulin (HG + HI) for 48 h. The cardiomyocytes were then incubation with SPRC (50 μM) for 24 h. ^3^H glucose uptake was measured in a liquid scintillation counter and the concentration of protein were detected by BCA method for standardization (*n* = 3). *P < 0.05 and ***P < 0.001 vs. control group, ^#^*P* < 0.05, ^*##*^*P* < 0.01 vs. HG+HI group. Values are presented as means ± SEM.

GLUT4 transporter is the most abundant glucose transporter in the heart, which is also the major glucose transporter translocating to the plasma membrane in response to insulin (22). As shown in Figures 8A,B, the protein expression level of GLUT4 were significantly decreased in diabetic heart, and SPRC treatment increased the mRNA and protein expression level of GLUT4. SPRC also enhanced the glucose uptake *in vitro* (Figure 8C).

### SPRC Treatment Increased CSE Expression and H_2_S Content in the Myocardium

**Figure 9:**
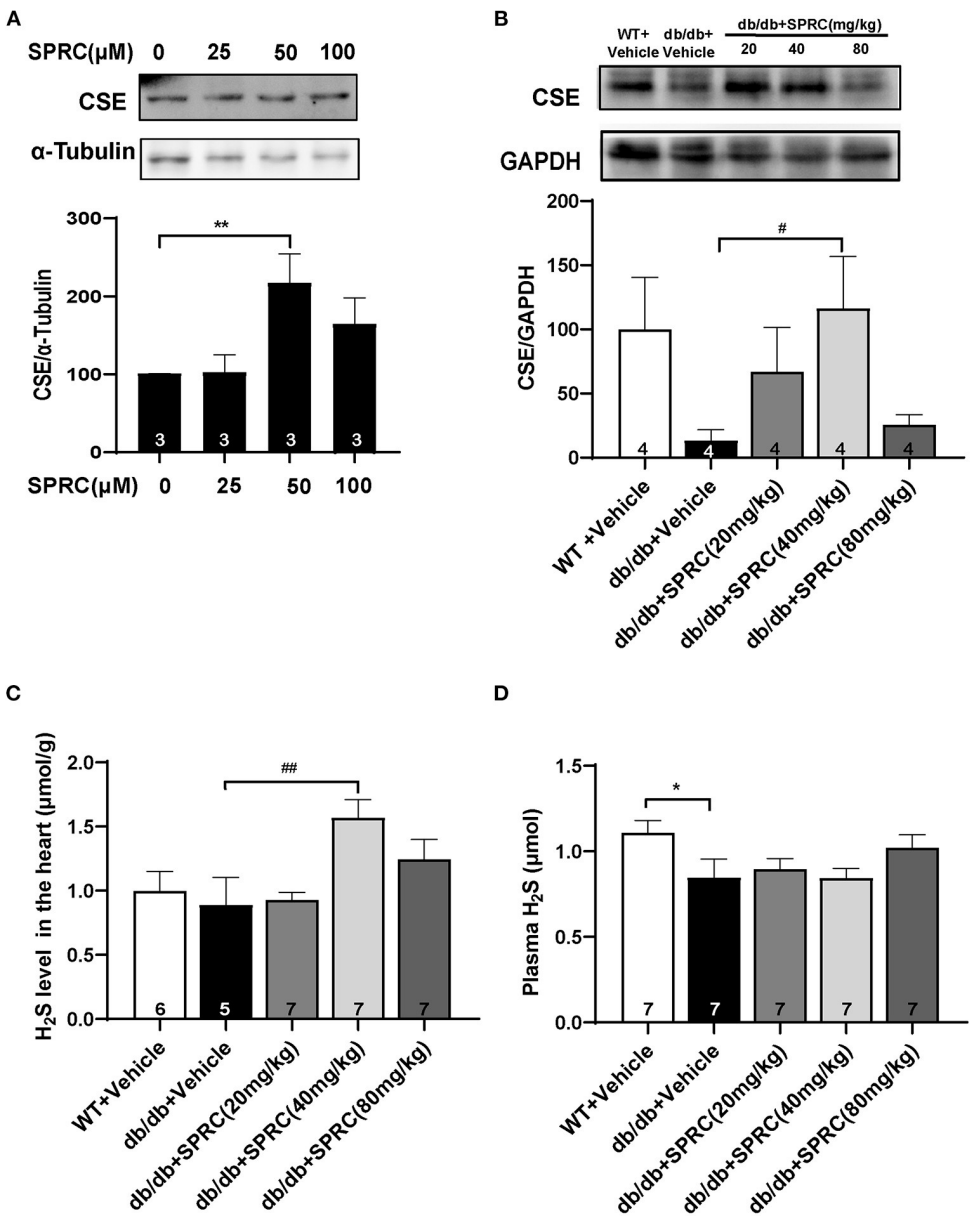
SPRC treatment increased CSE expression and H_2_S content in the myocardium. **(A)** Primary mice cardiomyocytes were treated with vehicle or SPRC (25, 50, and 100 μM) for 24 h. The expression level of CSE were determined by western blotting (*n* = 3). **P < 0.01 vs. Vehicle group. **(B–D)** Vehicle or SPRC (20, 40, or 80 mg/kg/day) was orally administered to WT mice or *db/db* mice for 12 weeks. The expression level of CSE were determined by western blotting (*n* = 4), H_2_S levels in the myocardium (*n* = 5–7), and plasma (*n* = 7) were detected. *P < 0.05, **P < 0.01 vs. WT + Vehicle group, ^#^*P* < 0.05, ^*##*^*P* < 0.01 vs. *db/db* + Vehicle mice. Values are presented as means ± SEM.

CSE is the primary H_2_S-generating enzyme in the cardiovascular system, catalyzing the synthesis of endogenous H_2_S from L-cysteine, and SPRC is an activator of CSE. We confirmed that SPRC increases the protein expression of CSE in primary cardiomyocytes (Figure 9A). Moreover, SPRC enhanced CSE expression in the left ventricular tissues of *db/db* mice (Figure 9B). Furthermore, H_2_S concentration in plasma and myocardium were detected. SPRC treatment at the doses of 40 and 80 mg·kg^−1^·day^−1^ improved H_2_S levels in the myocardium of *db/db* mice (Figure 9C). And H_2_S levels in plasma of *db/db* mice was significantly lower than that of WT mice, with SPRC treatment failed to rescue (Figure 9D).

## Discussion

In this study, we investigated the effects of SPRC on diabetic cardiomyopathy. Our results suggested that SPRC treatment could improve the myocardial function in diabetic mice with diabetic cardiomyopathy by attenuating myocardial hypertrophy, myocardial fibrosis, myocardial lipid accumulation, abnormalities in the ultrastructure of cardiomyocytes. Importantly, we unveiled the molecular mechanism underlying SPRC protected myocardium, which involves the activation of cardiac insulin receptor signaling.

The global prevalence of type 2 diabetes mellitus has been gradually increasing over half a century especially in developing countries (23). The systemic metabolic disorder in diabetic patients exposes myocardium to hyperglycemia, hyperinsulinemia, hyperlipidemia, and insulin resistance, all of which trigger the development of myocardial dysfunction. It is widely accepted that morphological phenotypes of diabetic cardiomyopathy include cardiac hypertrophy, cardiac fibrosis, increased intramyocardial lipids, while the functional phenotypes include left ventricular diastolic dysfunction which usually precede systolic dysfunction (24–26). In this study, we used spontaneously diabetic *db/db* mice as type 2 diabetes model which basically reflects all the manifestation of diabetic cardiomyopathy (18, 27). Moreover, 12 weeks of SPRC treatment, especially in the medium-dose group (40 mg·kg^−1^·day^−1^), alleviated almost all the morphological and functional phenotype of diabetic cardiomyopathy in *db/db* mice, which indicated the protective effect of SPRC on diabetic cardiomyopathy. Consistently, only the medium-dose group (40 mg·kg^−1^·day^−1^) had a significant increase in H_2_S levels in the myocardium (Figure 9C), while high-dose (80 mg·kg^−1^·day^−1^) SPRC only raised H_2_S levels in the myocardium a little and has no statistical significance when compared with vehicle. It has been reported that the mechanisms of SPRC releasing H_2_S include up-regulating CSE gene and protein expression (28, 29), as well as binding and activating CSE (16). Detailed mechanism of effective dose range of SPRC *in vivo* needs further study.

Next, we explored the mechanism of SPRC to protect diabetic cardiomyopathy. Abnormal mitochondrial morphology is common in the progression of diabetic cardiomyopathy, and suggests the possibility of impaired mitochondrial dynamics, metabolic substrate imbalance and increased oxidative stress (5, 30). Under transmission electron microscopy, we observed obvious damage of myocardial ultrastructure in *db/db* mice, especially alterations of mitochondria. SPRC treatment markedly restored mitochondrial and myocardial ultrastructure. This result suggests that the effect of SPRC on diabetic cardiomyopathy may associated with improvement of myocardial mitochondria. The effect of SPRC treatment on mitochondrial function, myocardial oxidative stress and underlying signaling pathway is worth of further study.

Under conventional conditions in heart, glucose and lactic acid only supply 30% of ATP generation, with the majority of ATP generated by fatty acid (FA) oxidation. Whereas, in the case of type 2 diabetes mellitus, hyperglycemia maintains cardiac glucose uptake, but the proportion of FA as a metabolic substrate further increases in consequence of plasma FA availability (31–35). Within the heart, lipid droplet accumulation generally only appears in patients with diabetes or metabolic syndrome, indicating an imbalance of FA uptake/synthesis and consumption (36, 37). In this study, both hyperlipidaemia and cardiac lipid droplet accumulation were observed in *db/db* mice, and the effect of SPRC treatment on improving lipid content in myocardium seems superior to the improvement of lipid content in circulation. Similarly, SPRC treatment protected diabetic mice from cardiac insulin resistance by activating the AKT/GLUT4 signaling, with no obvious improvement on systemic insulin resistance, which were consistent with the improvement of H_2_S levels in myocardium but not in plasma. This may be because *db/db* mice as transgenic rodent is completely resistant to leptin receptor, developed morbid obesity and severe systemic metabolic phenotype at 20 weeks of age which is irreversible by SPRC treatment (23). Besides, this also demonstrates that the protective effect of SPRC treatment is targeted to the myocardium, rather than the consequence of alleviating the metabolic phenotype of whole body. In addition, SPRC releases H_2_S *via* the up-regulating of CSE, thus the organ distribution of CSE is critical to the effect of SPRC. Next, we focus on the effect of SPRC on cardiac molecular signaling pathway in the following experiments.

Insulin resistance is defined as impaired insulin signaling together with diminution in glucose transport, which promotes the development of diabetic cardiomyopathy (38). Myocardial insulin signaling plays an important role in myocardial metabolic remodeling in response to myocardial metabolic disorders by regulating glucose uptake, long-chain fatty acid uptake, and protein synthesis, wherein insulin receptor (IR) is a key protein for signal transduction (39–41). Cardiomyocyte-selective insulin receptor knockout mice revealed decreased insulin signaling in cardiomyocytes without systemic metabolic disturbances, and showed worsen cardiac remodeling in respond to stress (42) and accelerated cardiac mitochondrial dysfunction after myocardial infarction (43).

IR is a polymer transmembrane glycoprotein composed of α_2_β_2_ heterotetramer and belongs to the receptor tyrosine kinases (RTK) family Type Ò subfamily activated by ligands such as insulin and insulin-like growth factor (IGF) (44). The binding of insulin triggers autophosphorylation in kinase activation region of IR at Tyr-1146 and either Tyr-1150 or Tyr-1151 (45). The activated IR recruits and phosphorylates scaffold proteins such as insulin receptor substrates (IRS) to activate the classical PI3K/AKT pathway, with other downstream pathway of insulin signaling (including Ras/MAPK-dependent pathways) unaffected. The activation of AKT subsequently promotes the translocation of GLUT4 to the membrane, and enhances glucose uptake. The phosphorylation of AKT also inactivates GSK-3β, an essential negative regulator of cardiac hypertrophy and cardiomyopathy (46, 47). Consistent with these studies, our results showed inhibited insulin signaling such as decreased phosphorylation levels of Akt and GSK-3β and reduced protein expression of GLUT4 in the cardiomyocyte of diabetic hearts, which was notably activated by SPRC treatment. Moreover, we speculate that SPRC enhanced the glucose uptake to regulate metabolic substrate proportion, thereby protecting the heart caused by metabolic disorders. Interestingly, although the effect of SPRC on IR downstream signaling *in vivo* and *in vitro* were consistent, the phosphorylation status of IR differs. The phosphorylation level of IR was reduced by acute high concentrations of glucose and insulin in *vitro*, but tends to increase in chronic diabetic myocardium which may due to compensation. This suggests that in the myocardium of type 2 diabetes, the inhibition of IR signaling is not due to the decrease of phosphorylation level of IR, but the decrease of IR expression and downstream signaling transduction.

In summary, we provided evidences that SPRC protects against cardiac fibrosis and improves myocardial function in diabetic mice. This mechanism involves increased expression and activity of IR and activated Akt/ GSK-3β signaling, which subsequently enhanced glucose uptake in cardiomyocyte, resulting in improved diabetic cardiomyopathy. Thereby, SPRC may be a promising medication for diabetic cardiomyopathy in type 2 diabetes mellitus patients.

## Data Availability Statement

The original contributions presented in the study are included in the article/Supplementary Material, further inquiries can be directed to the corresponding author/s.

## Ethics Statement

The animal study was reviewed and approved by The Ethics Committee of Experimental Research, Fudan University, Shanghai Medical College.

## Author Contributions

Y-CZ and YL: designed experiments. YL, K-FX, Y-HC, and CW: performed experiments. YL and K-FX: analyzed data. Y-CZ, YC, and M-JW: provided laboratory space, reagents, and technical support. YL: wrote the manuscript. M-JW: revised the manuscript. Y-CZ: supervised the study. All authors contributed to the article and approved the submitted version.

## Funding

This work was supported by the National Natural Science Foundation of China (NSFC) (31830042 and 81870212 to Y-CZ, 81970361 to M-JW), the National Key Science and Technology Project of China (2018YFC2000200/02), Macau Science and Technology Development fund (FDCT 0007/2019/AKP), and the funding of Innovative research team of high-level local universities in Shanghai and a key laboratory program of the Education Commission of Shanghai Municipality (ZDSYS14005 to Y-CZ).

## Conflict of Interest

The authors declare that the research was conducted in the absence of any commercial or financial relationships that could be construed as a potential conflict of interest.

## Publisher's Note

All claims expressed in this article are solely those of the authors and do not necessarily represent those of their affiliated organizations, or those of the publisher, the editors and the reviewers. Any product that may be evaluated in this article, or claim that may be made by its manufacturer, is not guaranteed or endorsed by the publisher.

## Abbreviations

Akt/PKB: protein kinase B
AUC: area under the curve
CSE (CTH): cystathionine-γ-lyase
DCM: diabetic cardiomyopathy
DM: diabetes mellitus
DT: deceleration time
ECL: enhanced chemiluminescence
EF: ejection fraction
FBG: fasting blood glucose
FINS: fasting plasma insulin
FS: fraction shortening
H_2_S: hydrogen sulfide
GLUT4: glucose transporter 4
GSK3β: glycogen synthase kinase 3β
HE: hematoxylin-eosin
HDL-C: high-density lipoprotein cholesterol
HOMA-IR: homeostasis model assessment-insulin resistance index
IPGTT: Intraperitoneal glucose tolerance test
IPITT: Intraperitoneal Insulin Tolerance Test
LDL-C: low-density lipoprotein cholesterol
SPRC: S-propargyl-cysteine
TEM: transmission electron microscope
TC: total cholesterol
TG: triglycerides.
