# Supplementary figures and images for: S-Propargyl-Cysteine Attenuates Diabetic Cardiomyopathy in db/db Mice Through Activation of Cardiac Insulin Receptor Signaling
Source: Front Cardiovasc Med. 2021 Sep 17;8:737191. doi: 10.3389/fcvm.2021.737191 (PMC8484714; doi:10.3389/fcvm.2021.737191)

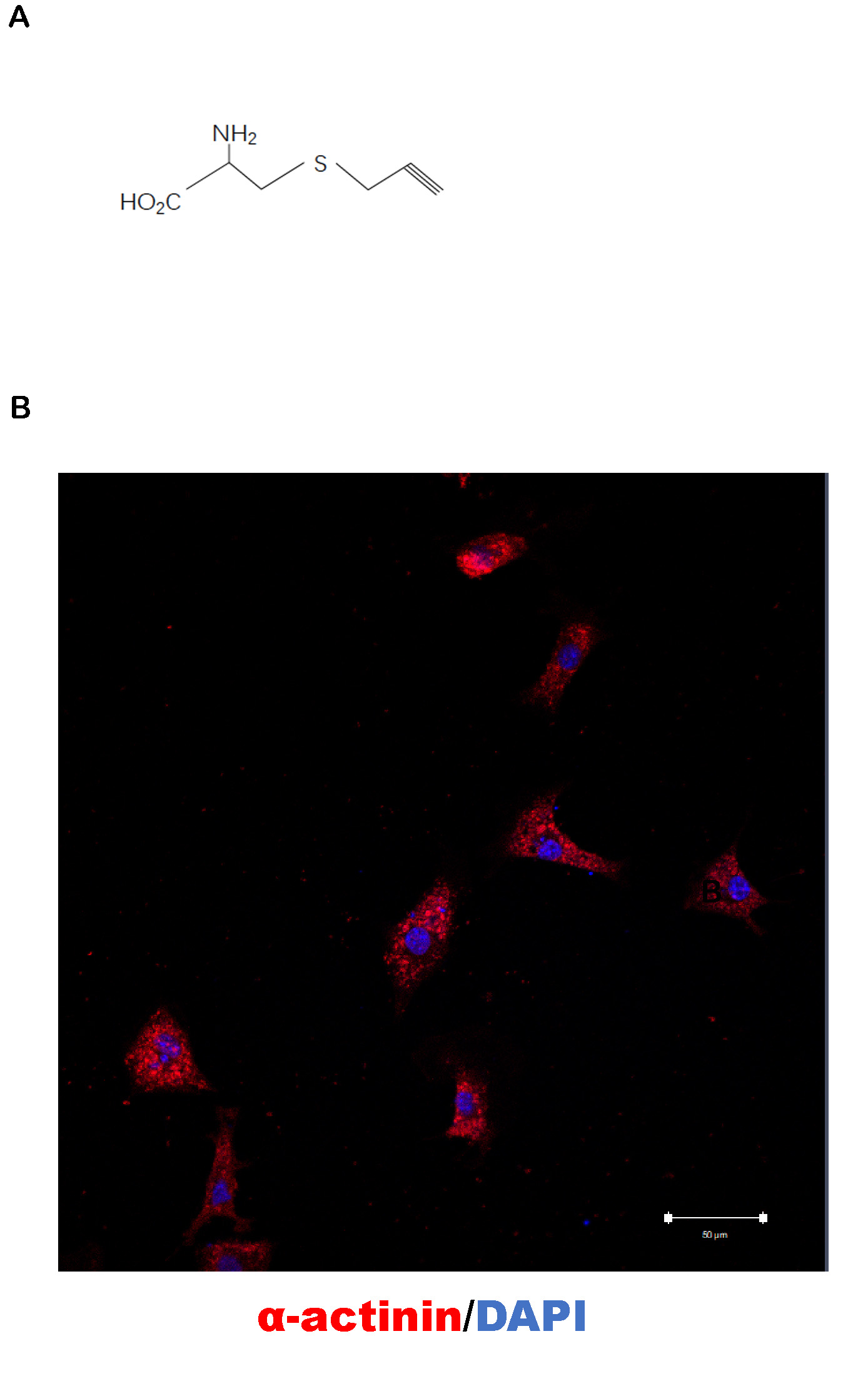

Supplement: Supplementary file 1 [file Image_1.jpg]
