# Supplementary material for: S-Propargyl-Cysteine Attenuates Diabetic Cardiomyopathy in db/db Mice Through Activation of Cardiac Insulin Receptor Signaling
Source: Front Cardiovasc Med. 2021 Sep 17;8:737191. doi: 10.3389/fcvm.2021.737191 (PMC8484714; doi:10.3389/fcvm.2021.737191)
Supplement: Supplementary file 2 [file Table_1.docx]

Table 1. Echocardiography data of mice at 8-week-old (Mean ± SEM)

|  | WT | *db/db* | *db/db* + SPRC (20 mg/kg) | *db/db* + SPRC (40 mg/kg) | *db/db* + SPRC (80mg/kg) |
| --- | --- | --- | --- | --- | --- |
| IVCT (ms) | 21.62 ± 3.2 | 14.1 ± 2.77 | 15.94 ± 4.18 | 12.21 ± 1.72 | 14.33 ± 0.97 |
| IVRT (ms) | 15.77 ± 2.57 | 20.14 ± 2.49 | 22.32 ± 1.84 | 21.3 ± 1.87 | 19.66 ± 4.57 |
| MV Decel (ms) | 26.83 ± 2.39 | 29.17 ± 3.81 | 32.56 ± 2.79 | 25.13 ± 2.54 | 31.89 ± 2.59 |
| MV A (mm/s) | 443.66 ± 48.54 | 471.46 ± 41.77 | 431.1 ± 61.38 | 362.38 ± 75.19 | 466.41 ± 32.84 |
| MV E (mm/s) | 746.56 ± 55.26 | 723.85 ± 52.91 | 743.22 ± 51.97 | 736.98 ± 80.21 | 639.83 ± 37.34 |
| E/A | 1.79 ± 0.27 | 1.64 ± 0.22 | 2.04 ± 0.56 | 1.81 ± 0.29 | 1.4 ± 0.18 |
| MV PHT (ms) | 7.78 ± 0.69 | 8.46 ± 1.1 | 9.44 ± 0.81 | 7.29 ± 0.74 | 9.25 ± 0.75 |
| IVS; d (mm) | 1.00 ± 0.07 | 0.93 ± 0.1 | 0.83 ± 0.05 | 0.92 ± 0.04 | 0.96 ± 0.08 |
| IVS; s (mm) | 1.21 ± 0.05 | 1.06 ± 0.07 | 1.09 ± 0.07 | 1.19 ± 0.05 | 1.15 ± 0.06 |
| LVID; d (mm) | 3.69 ± 0.17 | 4.02 ± 0.1 | 4.24 ± 0.19 | 4.16 ± 0.18 | 4.13 ± 0.19 |
| LVID; s (mm) | 2.39 ± 0.18 | 2.88 ± 0.19 | 3.09 ± 0.2 | 2.95 ± 0.22 | 2.97 ± 0.2 |
| LVPW; d (mm) | 1.14 ± 0.06 | 0.97 ± 0.09 | 0.83 ± 0.12 | 0.86 ± 0.02 | 0.98 ± 0.07 |
| LVPW; s (mm) | 1.47 ± 0.08 | 1.28 ± 0.11 | 1.02 ± 0.12 | 1.1 ± 0.04 | 0.97 ± 0.16 |
| LV Mass (mg) | 125.58 ± 11.78 | 122.73 ± 17.03 | 109.62 ± 9.15 | 116.45 ± 8.88 | 128.43 ± 7.41 |
| LV Vol; d (μl) | 58.58 ± 6.44 | 71.07 ± 3.85 | 81.24 ± 8.26 | 77.99 ± 7.45 | 76.19 ± 7.86 |
| LV Vol; s (μl) | 20.84 ± 3.79 | 32.47 ± 4.87 | 38.79 ± 5.5 | 35.04 ± 5.79 | 34.78 ± 5.08 |
| Heart Rate (BPM) | 451.62 ± 24.02 | 421.37 ± 17.72 | 416.48 ± 15.12 | 430.31 ± 13.46 | 452.22 ± 15.11 |
| Stroke Volume (μl) | 38.01 ± 3.06 | 39.81 ± 2.6 | 43.76 ± 4.53 | 45.08 ± 3.19 | 43.84 ± 4.6 |
| EF (%) | 67.72 ± 3.47 | 58.34 ± 3.2 | 55.16 ± 3.29 | 58.74 ± 4.17 | 58.79 ± 2.33 |
| FS % | 37.34 ± 2.58 | 30.48 ± 2.02 | 28.55 ± 2.12 | 31.14 ± 2.86 | 30.8 ± 1.49 |
| Cardiac Output (mL/min) | 17.31 ± 1.94 | 16.89 ± 1.57 | 18 ± 1.41 | 19.4 ± 1.51 | 19.96 ± 2.51 |

Table 2. Echocardiography data of mice at 20-week-old (Mean ± SEM)

|  | WT | *db/db* | *db/db* + SPRC (20 mg/kg) | *db/db* + SPRC (40 mg/kg) | *db/db* + SPRC (80mg/kg) |
| --- | --- | --- | --- | --- | --- |
| AET (ms) | 47.1 ± 1.76 | 52.74 ± 2.37 | 53.62 ± 2.61 | 55.03 ± 5.68 | 54.13 ± 1.46 |
| IVCT (ms) | 11.86 ± 1.13 | 12.01 ± 1.51 | 10.64 ± 1.4 | 10.11 ± 1.37 | 13.71 ± 1.06 |
| IVRT (ms) | 13.7 ± 1.02 | 16.7 ± 1.63 | 17.67 ± 1.84 | 13.29 ± 1.24 | 18.11 ± 1.25 |
| MV Decel (ms) | 23.7 ± 2.39 | 37.27 ± 1.65^***^ | 34.19 ± 2.63 | 32.08 ± 1.8 | 24.13 ± 1.26^###^ |
| MV A (mm/s) | 389.09 ± 12.12 | 421.85 ± 18.15 | 409.19 ± 25.41 | 384.12 ± 23.46 | 415.6 ± 26.86 |
| MV E (mm/s) | 633.85 ± 27.6 | 654.55 ± 60.93 | 642.83 ± 48.6 | 624.26 ± 46.84 | 631.7 ± 35.81 |
| E/A | 1.64 ± 0.09 | 1.54 ± 0.11 | 1.63 ± 0.16 | 1.62 ± 0.05 | 1.59 ± 0.14 |
| MV PHT (ms) | 6.87 ± 0.69 | 10.81 ± 0.48^***^ | 9.91 ± 0.76 | 9.3 ± 0.52 | 7 ± 0.36^###^ |
| IVS; d (mm) | 0.96 ± 0.1 | 1.12 ± 0.05 | 1.23 ± 0.07 | 1.22 ± 0.1 | 1.14 ± 0.04 |
| IVS; s (mm) | 1.2 ± 0.12 | 1.5 ± 0.07^*^ | 1.61 ± 0.06 | 1.51 ± 0.11 | 1.47 ± 0.05 |
| LVID; d (mm) | 3.81 ± 0.13 | 3.97 ± 0.13 | 3.66 ± 0.16 | 3.66 ± 0.2 | 3.74 ± 0.11 |
| LVID; s (mm) | 2.45 ± 0.11 | 2.68 ± 0.11 | 2.47 ± 0.17 | 2.31 ± 0.16^#^ | 2.41 ± 0.1 |
| LVPW; d (mm) | 0.81 ± 0.05 | 0.61 ± 0.04 | 0.71 ± 0.1 | 0.78 ± 0.12 | 0.68 ± 0.07 |
| LVPW; s (mm) | 1.09 ± 0.09 | 0.75 ± 0.03 | 0.89 ± 0.1 | 1.16 ± 0.16 | 0.93 ± 0.09 |
| LV Mass (mg) | 109.71 ± 4.2 | 102.66 ± 7.61 | 105.63 ± 3.19 | 111.22 ± 4.21 | 111.5 ± 9.16 |
| LV Vol; d (μl) | 67.1 ± 4.53 | 69.7 ± 5.77 | 57.57 ± 8.01 | 57.91 ± 9.62 | 68.73 ± 3.99 |
| LV Vol; s (μl) | 22.91 ± 2.62 | 27.12 ± 2.98 | 21.19 ± 3.21 | 17.53 ± 3.18^#^ | 23.67 ± 2.88 |
| Heart Rate (BPM) | 458.43 ± 7.34 | 470.23 ± 7.13 | 457.9 ± 10.84 | 469.26 ± 8.11 | 463.19 ± 9.44 |
| Stroke Volume (μl) | 41.97 ± 2.82 | 40.17±3.14 | 35.28 ± 2.54 | 38.37 ± 3.2 | 36.99 ± 3.01 |
| EF (%) | 65.68 ± 1.94 | 59.45 ± 1.39^*^ | 61.99 ± 2.33 | 67.02 ± 2.85^#^ | 63.73 ± 2.8 |
| FS % | 35.73±1.53 | 31.18 ± 0.97 | 32.97 ± 1.63 | 36.8 ± 2.24^#^ | 34.36 ± 2.17 |
| Cardiac Output (mL/min) | 20.84 ± 1.27 | 18.92 ± 1.55 | 16.07 ± 1.02 | 17.89 ± 1.32 | 17.02 ± 1.31 |

AET, Aortic ejection time; IVCT, Isovolumic contraction time; IVRT, Isovolumic relaxation time; MV Decel, Mitral valve deceleration time; PHT, Pressure half-time; HR, heart rate; IVSd, interventricular septum at diastole; IVSs, interventricular septum at systole; LVIDd, LV internal diameter at diastole; LVIDs, LV internal diameter at systole; LVPWd, LV posterior wall thickness at diastole; LVPWs, LV posterior wall thickness at systole; SV, stroke volume; CO, cardiac output; EF, ejection fraction; FS, fractional shortening; ****P < 0.05, ***P < 0.001 versus WT mice, #P < 0.05, ###P < 0.001 versus db/db mice.***
